# Coiled-Coil Protein Hydrogels Engineered with Minimized Fiber Diameters for Sustained Release of Doxorubicin in Triple-Negative Breast Cancer

**DOI:** 10.1021/acsbiomaterials.4c00349

**Published:** 2024-04-15

**Authors:** Dustin Britton, Jakub Legocki, Deven Paul, Olga Katsara, Orlando Aristizabal, Neelam Pandya, Orin Mishkit, Yingxin Xiao, Matias Aristizabal, Neha Rahman, Robert Schneider, Youssef Z. Wadghiri, Jin Kim Montclare

**Affiliations:** † Department of Chemical and Biomolecular Engineering, New York University Tandon School of Engineering, Brooklyn, New York 11201, United States; ∥ Department of Microbiology, New York University Grossman School of Medicine, New York, New York 10016, United States; § Center for Advanced Imaging Innovation and Research (CAI2R), New York University Grossman School of Medicine, New York, New York 10016, United States; ¶ Bernard and Irene Schwartz Center for Biomedical Imaging, Department of Radiology, New York University Grossman School of Medicine, New York, New York 10016, United States; £ Department of Biomedical Engineering, New York University Tandon School of Engineering, Brooklyn ,New York11201, United States; # Department of Radiation Oncology, New York University Grossman School of Medicine, New York, New York 10016, United States; ∇ Department of Chemistry, New York University, New York, New York 10012, United States; ° Department of Biomaterials, New York University College of Dentistry, New York, New York 10010, United States

**Keywords:** protein engineering, hydrogel, computational design, drug delivery, triple negative breast cancer

## Abstract

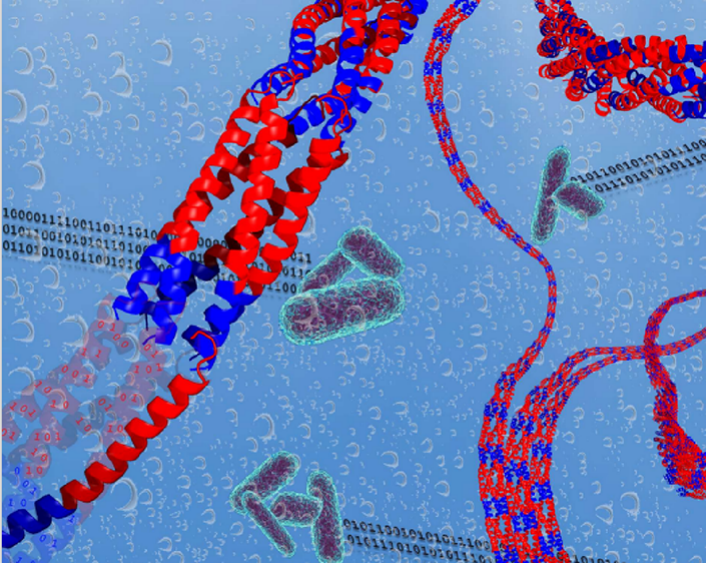

Triple-negative breast cancer (TNBC) lacks expressed protein targets, making therapy development challenging. Hydrogels offer a promising new route in this regard by improving the chemotherapeutic efficacy through increased solubility and sustained release. Moreover, subcutaneous hydrogel administration reduces patient burden by requiring less therapy and shorter treatment times. We recently established the design principles for the supramolecular assembly of single-domain coiled-coils into hydrogels. Using a modified computational design algorithm, we designed Q8, a hydrogel with rapid assembly for faster therapeutic hydrogel preparation. Q8 encapsulates and releases doxorubicin (Dox), enabling localized sustained release via subcutaneous injection. Remarkably, a single subcutaneous injection of Dox-laden Q8 (Q8•Dox) significantly suppresses tumors within just 1 week. This work showcases the bottom-up engineering of a fully protein-based drug delivery vehicle for improved TBNC treatment via noninvasive localized therapy.

## Introduction

Triple negative breast cancer (TNBC) lacks the receptor biomarkers found in other subtypes, making treatment challenging.^1^ Unlike hormone receptor (HR)-positive, human epidermal growth factor receptor 2 (HER2)-positive, estrogen receptor (ER), and progesterone receptor (PR) breast cancers, TBNC does not respond to targeted therapies,^2,3^ leaving chemotherapy as the primary option.^4,5^

Cytotoxic chemotherapy is the predominant treatment of early- and late-stage TNBC,^4,5^ but its efficacy is hampered by limitations like poor bioavailability and resistance. There has been significant research interest in improving the efficacy of the treatment via enhancing its bioavailability.^6^ It becomes increasingly important to provide increased delivery or targeting of chemotherapy in consideration of cancer chemoresistance, which is a leading cause for cancer recurrence.^7^ To overcome these issues, various biocompatible materials^6,8^ like lipids,^9,10^ polymers,^11−14^ and proteins^15−17^ have been developed as drug delivery carriers to encapsulate a chemotherapeutic and enhance its circulation and selective delivery. These materials often take the form of nanoparticles or hydrogel-based systems. Whereas the former can suffer from low biocompatibility and nonspecific accumulation,^18^ hydrogel-based systems offer a biocompatible drug delivery method for controlled and localized release that allows for minimized drug content and systemic toxicity.^19,20^

Several macroscopic hydrogels—for *in situ* implantation/injection or transdermal delivery—have been developed for improved chemotherapeutic efficacy.^21^ In cases that bypass surgical intervention via *in situ* injection, researchers have relied on self-assembled or cross-linked polymer or polymer-hybrid systems and have used targeting moieties and loaded immunotherapeutics to improve their efficacy.^22−26^ Conversely, the use of proteins as drug delivery vehicles benefit from a modular amino acid sequence,^27,28^ which offers the ability to tune the mechanical properties and drug encapsulation and release of a chemotherapeutic.^29,30^

We have recently discovered that the coiled-coil protein hydrogel supramolecular assembly depends on their surface-facing electrostatic interactions between positively and negatively charged N- and C-termini (ΔEE_bcf_).^30^ Furthermore, we have shown the ability to predict and design hydrogels for increased gelation rates and mechanical strength^29,31^ based on tuning the electrostatic potentials and Rosetta score by employing a trimodal Monte Carlo search toward the minimum Rosetta Score and specific Δ*E*E_bcf_.^29^ Previously, we have demonstrated that tuning a coiled-coil hydrogel with improved mechanical strength also possesses improved hydrophobic small molecule encapsulation.^31^ Moreover, improved release has been previously attributed to improved mechanical strength, which confers an increased chance of collision between drugs and slower diffusion.^32,33^

Here, we modified our trimodal Monte Carlo search to minimize the ΔEE_bcf_ and the fiber diameters of supramolecular assembling hydrogels. Our resulting protein hydrogel, Q8, possesses a 2-fold increase in both gelation rate and mechanical strength compared to our previously measured fastest gelation times and storage moduli in Q5 and Q7, respectively.^29^ Having demonstrated improved mechanical properties, we study Q8 as the first completely protein-based macroscopic hydrogel for sustained chemotherapeutic delivery to tumor mouse models *in vivo* where doxorubicin delivered in Q8 exhibits significantly improved tumor suppression compared to doxorubicin alone using a single subcutaneous injection for noninvasive treatment of TNBC.

## Materials and Methods

### Materials

Chemically competent M15MA *E. coli* cells were gifted from David Tirrell at California Institute of Technology.^34^ Bacto-tryptone, sodium chloride (NaCl), yeast extract, tryptic soy agar, ampicillin sodium salt, sodium phosphate dibasic anhydrous (Na_2_HPO_4_), sodium hydroxide (NaOH), dextrose monohydrate (d-glucose), magnesium sulfate (MgSO_4_), calcium chloride (CaCl_2_), manganese chloride tetrahydrate (MnCl_2_·4H_2_O), cobaltous chloride hexahydrate (CoCl_2_·6H_2_O), isopropyl-β-d-1-thiogalactopyranoside (IPTG), Pierce bicinchoninic acid (BCA) assay kit, Pierce snakeskin dialysis tubing 3.5K molecular weight cutoff (MWCO), sodium dodecyl sulfate (SDS), Nunc 96-well plates, Molecular Probes FluoSpheres (1.0 μm), and BD Clay Adams glass microscopy slides were acquired from Thermo Fisher Scientific (Rochester, NY, USA). The 20 naturally occurring amino acids and thiamine hydrochloride (vitamin B) were purchased from Sigma-Aldrich. Hydrochloric acid (HCl) and Coomassie Brilliant Blue G-250 were purchased from VWR (Bridgeport, NJ, USA). HiTrap FF 5 mL columns for protein purification were purchased from Cytiva Life Sciences. Macrosep and Microsep Advance Centrifugal Devices 3K MWCO and 0.2 μm syringe filters were purchased from PALL. Acrylamide/bis solution (30%) 29:1 and natural polypeptide sodium dodecyl sulfate–polyacrylamide gel electrophoresis (SDS-PAGE) standard were purchased from Bio-Rad. Imidazole was purchased from Acros Organics (Rochester, NY, USA). Formvar/carbon-coated copper grids (FCF400-Cu) and 1% uranyl acetate for transmission electron microscopy were purchased from Electron Microscopy Sciences. Borosilicate glass capillaries (0.2 × 2 × 75 mm) were purchased from VitroCom (Mountain Lakes, NJ, USA). Fast-curing two-component epoxy was acquired from JB Weld. 4T1 cells (CRL-2539) were purchased from ATCC. Balb/cJ mice (000651) were purchased from Jackson Laboratories (Bar Harbor, ME, USA). Dulbecco’s modified Eagle's medium (DMEM, 10-013-CV), trypsin (25-053-CI), phosphate-buffered saline (PBS, 21-040-CM), and Matrigel (356237) were purchased from Corning (Corning, NY, USA). Fetal bovine serum (FBS) was from Laboratory Disposable Products (Wayne, NJ, USA).

### Computational Design of Q8

Q8 was designed using a modified version of a trimodal Monte Carlo Search developed previously.^29^ The Rosetta suite of macromolecular modeling tools (Version 3.5) was used to model protein mutants and calculate Rosetta scores, with lower energy scores indicating higher stability, using the Rosetta Relax protocol^35^ with the all-atom energy score function.^36^ PDB2QR and APBS^37^ were used to calculate the electrostatic potential of surface residues, EE_bcf_, for the N- and C-terminus (NE_bcf_ and CE_bcf_) and were subsequently used to calculate the difference of the N- and C-terminal EE_bcf_, dubbed ΔEE_bcf_.^30^ Important to note is that all computational modeling excluded the His-tag region (AA 1–16) because the structural prediction of the region lacks confidence due to its random coil secondary structure.^29−31,38^ Thus, Rosetta score values and resulting NE_bcf_ values were calculated in the absence of the His-tag region, and these values are otherwise considered a constant in comparison of different variants. The total probability of selecting a mutant with a worse (higher) Rosetta score (*P*_RS_) (eq 1) or worse (higher) NE_bcf_ or CE_bcf_ (eq 2) was used as a criterion for making a mutation in Rosetta. The script was also modified to allow Rosetta to make mutations to the *a* and *d* helical wheel positions from the following residue list: V, I, M, T, Q, and L based on the likelihood for homopentameric coiled-coils.^39^ Charged or neutral mutations were allowed for the *b, c, e, f,* and *g* helical wheel positions from the following residue list: A, E, K, Q, N, T, and D.
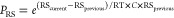
1
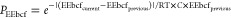
2

Here, RS is the Rosetta score [J/mol], RT [J/mol] is the product of the molar gas constant and temperature, and *C* is an empirical constant used to constrain the probability criteria during the search. A *C* value of 3.93 × 10^–5^ [mol/J] was used in eq 1, and a *C* value of 1.31 × 10^–4^ and 1.96 × 10^–4^ [mol/J] was used in eq 2 in our searches for N- and C-terminal EE_bcf_, respectively. The final protein structures were visualized using PyMOL (Schrodinger, LLC., Version 2.5.4)^40^ with the APBS plugin.^37^

### Computational Doxorubicin Docking

Doxorubicin (Dox) was assessed for encapsulation in the hydrophobic pore of Q8 using the Rosetta GALigand Docking protocol^41^ based on a high-throughput screening model developed previously.^42^ The Dox chemical structure was sourced from DrugBank,^43^ and OpenEye OMEGA^44^ was used to establish minimum energy conformers prior to docking. Dox was placed near the N-terminus and C-terminus of the coiled-coil to assist in covering the entire distance of the coiled-coil pore. For each placement, 1000 poses were generated, and interface scores were used to determine the best docked structures.

### Protein Expression

Q8 protein was expressed as described previously.^29^ Briefly, the Q8/pQE60 plasmid, purchased from Genscript, was transformed into chemical competent M15MA *E. coli* cells on tryptic soy agar plates. Colonies were used to inoculate 16 mL of starter cultures composed of supplemented M9 media, which were subsequently used to inoculate 400 mL of supplemented M9 media after overnight incubation. IPTG (200 μg/mL) was used to induce expression when the optical density at 600 nm (OD_600_) grew to 0.8–1.0. Expression was allowed for 3 h at 37 °C and 350 rpm before cells were harvested by centrifugation at 5000*g* at 4 °C for 20 min in an Avanti J-25 centrifuge (Beckman Coulter, Brea, CA, USA), and pellets were stored at −20 °C until purification.

### Protein Purification

Q8 protein was purified as described previously.^29^ First, pellets were lysed in 40 mL of buffer A (50 mM Tris-HCl and 500 mM NaCl; pH 8.0) using a Q500 probe sonicator (QSonica) at 55% amplitude for 2 min with 5 s on and 5 s off pulses on ice. The cell lysate was separated by centrifugation at 11,000*g* for 50 min and flown through a cobalt-charged HiTrap IMAC FF 5 mL column. The protein was eluted by flowing through increasing concentrations of buffer B (50 mM Tris-HCl, 500 mM NaCl, and 500 mM imidazole). Fractions were assessed for purity by 12% SDS-PAGE (Figure S1a), and pure fractions were dialyzed using six consecutive buckets of buffer A at 5 L volumes in 3.5 kDa MWCO snakeskin tubing at room temperature (Figure S1b). The His-tag of the protein was retained following purification, as done with previous Q protein variants. Because the His-tag provides a large contribution to the large N-terminal electrostatic potential, it is considered critical to the supramolecular assembly and is considered in the predictive modeling of ΔEE_bcf_.^29^

### Protein Concentration

The desired protein concentration was achieved by using 3 kDa MWCO Macrosep and Microsep Advance centrifugal devices (Pall Corporation) within 6 h of removal from dialysis bags. The protein concentration was determined by the bicinchoninic acid (BCA) assay with a standard curve made using dilutions of bovine serum albumin (BSA).

### Doxorubicin Loading and Release

Dox loading was performed consistent with the previous Q hydrogel drug loading of curcumin.^31,45^ Fresh anhydrous Dox was prepared at 1 or 3 mM final concentrations in 50 mM TrisHCl and 500 mM NaCl (pH 8.0) buffer with a final 5% DMSO volume. Dox (300 μL) was then pipetted on top of the hydrogel (2 and 3 mM), which remained phase separated, and was allowed to diffuse for 24 h into the Q8 hydrogel at 4 °C. The remaining Dox atop the hydrogel in solution phase was pipetted out, and two additional buffer washes using 300 μL of 50 mM TrisHCl and 500 mM NaCl (pH 8.0) buffer were used to wash out the remaining Dox and collected. The removed Dox and Dox washes were read for relative absorbance at 490 nm and compared to a standard curve using the original Dox stock samples to determine their relative concentrations and subsequently used to calculate the total Dox loaded in the hydrogel. Dox release was also performed consistent with the previous Q hydrogel release of curcumin.^45^ Following Dox loading, Q8•Dox was assessed for release kinetics by allowing for incubation at 37 °C and 300 rpm (Thermomixer R, Eppendorf) with 300 μL of 50 mM Tris and 500 mM NaCl (pH 8.0) with 5% v/v DMSO pipetted atop the hydrogel. Periodically, samples were removed and lightly centrifuged at 2500 rpm for 2.5 min, and the supernatant was collected and assessed spectrophotometrically for Dox and protein concentration by the BCA assay. The removed supernatant was then replaced with a new buffer, and the incubation was allowed to continue. The experiment was ended when the spectrophotometric signal for Dox or protein was no longer observed.

### Microrheology

Gelation kinetics of Q8 was assessed using a microrheological assay as described previously.^46^ Briefly, immediately after the Q8 samples were concentrated to 2 mM, 30 μL aliquots were mixed with 1% v/v 1 μm diameter FluoSpheres inside a glass capillary tube (VitroCom). Samples were imaged periodically using an inverted fluorescent microscope (ZEISS Microscopy) at 40× magnification with 2 × 2 binning periodically while being incubated at 4 °C on a rotisserie at 8 rpm between measurements. Relaxation exponents were tracked until a negligible difference was observed with the relaxation exponent of the previous time point. Images were stacked, converted to grayscale, and analyzed with multiple particle tracking (MPT) in MATLAB (Mathworks, R2021a) using a code developed in-house and originally developed and modified by Dufresne, Kilfoil, Blair, and O’Neill as done previously.^46^

### Tube Inversion

Transition from solution-like to gel-like behavior was assessed using tube inversion as done for previous iterations of Q variants.^29,31,45,47^ Tube inversion was also used to generate a phase diagram by assessing binary tube inversion success at a variety of concentration and temperature incubation pairings.^29,31^

### Rheology

Parallel plate rheometry was used to assess the mechanical strength of Q8 hydrogels using a stress-controlled rheometer (Discovery Hybrid Rheometer 2, TA Instruments) equipped with parallel plate geometry. After gelation was confirmed by microrheology, Q8 hydrogels were loaded onto an 8 mm diameter lower and upper plate with a 0.2 mm geometry gap. Storage modulus (*G*′) and loss modulus (*G*′′) were measured from 0.1 to 10 Hz with 5% oscillation strain.^48^

### Circular Dichroism Spectroscopy

The protein secondary structure of Q8 was assessed in the solution state (prior to incubation at 4 °C) and in the gel state (after gelation was confirmed by microrheology after incubation at 4 °C) using circular dichroism (CD) spectroscopy. Spectra were measured using a Jasco J-815 CD spectrometer with a PTC-423S single position Peltier temperature control system of 15 μM Q8 diluted in water to minimize salt interference. The secondary structure of the solution state was measured immediately after concentration to 2 mM. Wavelength scans were performed from 195 to 250 at 1 nm step sizes, and mean residue ellipticity (MRE) was calculated as described previously.^49^

### Attenuated Total Reflectance-Fourier Transform Infrared Spectroscopy

The protein secondary structure of Q8 was assessed in the solution state (prior to incubation at 4 °C) and in the gel state (after confirming gelation by microrheology after incubation at 4 °C) using peak deconvolution of attenuated total reflectance-Fourier transform infrared (ATR-FTIR) spectra. Five microliters of the protein sample at 2 mM was allowed to rest for 1 min on a diamond crystal using a Nicolet 6700 Fourier transform infrared spectrometer equipped with a mercury cadmium telluride (MCT)-A detector. Spectra were collected from 4000 to 400 cm^–1^ with a 4.0 cm^–1^ resolution, normalized, and buffer-subtracted prior to analysis from 1700 to 1600 cm^–1^, corresponding to the amide I region.^50^ ATR-FTIR measurements were performed immediately after concentration to 2 mM for solution measurements. Peaks were deconvoluted using Gaussian functions in the PeakFit software until the goodness of fit reached *r*^2^ ≥ 0.99.^51,52^

### Transmission Electron Microscopy

Transmission electron microscopy (TEM) was performed as done previously^29,31^ to assess hydrogel fiber diameter, morphology, and cross-linking. TEM images were taken with an FEI Talos L120C transmission electron microscope using samples on a Formvar/carbon-coated copper grid. Samples were diluted to 50 μM, and 3 μL was spotted on the grids followed by a 5 μL wash with water and 3 μL staining with 1% v/v uranyl acetate solution each with incubation times of 1 min using a filter paper to gently wick the grids dry. Following imaging, minimum diameter nanofibers within the physically cross-linked hydrogel were sized in the ImageJ software (Version 1.52q).^53^

### Tumor Induction in Mice

All studies were approved by the NYU Grossman School of Medicine Institutional Animal Care and Use Committee (IACUC) and conducted in accordance with the IACUC guidelines. Female 6–8 week old Balb/cJ mice were used for murine 4T1 tumor studies (Jackson Laboratories, Bar Harbor, ME).

Mice received orthotopic injections in the fourth mammary fat pad with 3 × 10^5^ 4T1 cells, grown as previously described,^54^ in a total volume of 100 μL, including 30% Matrigel matrix (Corning). Tumor growth curves were obtained by measuring the tumor volumes by using an external caliper. Treatment groups, consisting of Dox, Q8, and Q8•Dox, were randomized upon tumor establishment at day 10 when the tumor reached an ∼100 mm^3^ volume. Treatment administrations were performed subcutaneously in the direct tumor proximity or systemically (intracardial injection). Throughout the trial, mouse tumor volumes were monitored using precision calipers every day for a week, when mice were sacrificed.^54^

### Ultrasound Imaging and Guided Injection

The high-frequency ultrasound (US) imaging was performed on a Vevo 3100 high-frequency ultrasound (US) system (Visualsonics/Fujifilm, Toronto, ON, CA). The system was equipped with an adjustable rail system designed for small animal handling, precise positioning, and optimization. This configuration facilitated noninvasive, *in vivo* imaging under accurate physiological conditions including a temperature-controlled heated stage, gas anesthesia, and a syringe injection system for simultaneous compound administration. A 50 MHz high-frequency US transducer (MX700 D) with an axial resolution of 30 μm and real-time imaging capability of up to 300 frames/s was employed.

### Image-Guided Intracardiac Infusion of Doxorubicin

Prior to the procedure, mice were initially anesthetized with 5.0% isoflurane in air and kept under anesthesia (1–2%) throughout Dox administration. For optimal imaging conditions, mice were positioned supine and immobilized by tapping the four paws to the conductive surfaces of the heated stage, enabling continuous electrocardiogram (ECG) monitoring. Body temperature was maintained at 35–37 °C. The chest fur was removed with a depilator agent. Ultrasound gel was applied over the precordial region for the visualization of the left ventricle. Once the cross-section with the largest left-ventricular chamber dimension was identified, a 1 mL hypodermic syringe (Becton Dickinson, UT, USA) equipped with a 30G needle (BD Insyte, 1 in. length, Becton Dickinson, UT, USA) was positioned on the chest wall, with the needle’s longitudinal axis aligned with the ultrasound imaging plane. Following the protocol established by Morsi et al.,^55^ real-time ultrasound-guided needle insertion facilitated puncture into the left ventricle followed by administration of a single injection of the 1 mM Dox solution, with a volume of 100 μL, over 90 to 120 s. This was repeated for five independent mice.

### Ultrasound Image-Guided Subcutaneous Delivery of Q8 and Q8•Dox Hydrogel

Q8 and Q8•Dox samples for ultrasound image-guided subcutaneous injections were prepared by loading a 1 mL syringe with freshly concentrated Q8 protein solution (3 mM, 100 μL) by using a rubber stopper to cap the opening. The syringes, loaded with Q8 protein, were allowed to incubate overnight at 4 °C to allow for transition into a hydrogel. For Q8•Dox samples, Dox solutions were freshly prepared and loaded atop the Q8 hydrogel to allow for 24 h diffusion before being washed of excess Dox, as described in Doxorubicin Loading and Release.

Prior to the intracutaneous injection of Q8 over the tumor, the ultrasound stage was positioned to facilitate access to the lower abdominal fat pad. Sterile ultrasound (US) gel was applied over the shaved fat pad area to enhance the visualization and guidance during the injection process. The US transducer was positioned perpendicular to the lower abdomen, providing a clear visualization of distinct echogenic multilayers. For the injection, a 30 gauge (30G) needle was carefully inserted laterally within the intracutaneous space above the tumor mass region. The Q8 solution (3 mM, 100 μL) was slowly infused through the needle within the intracutaneous space while continuously monitoring delivery over the tumor using ultrasound imaging. This was repeated for five independent mice for both the Q8 and Q8•Dox groups.

### *In vivo* Monitoring of Tumor Growth and Volume

To assess response to anticancer treatment, *in vivo* longitudinal monitoring of tumor growth and volume was performed using external caliper measurements. Seven days after the implantation of tumor cells, mice were monitored daily to assess their health and tumor growth through palpation of the injected fat pad until reaching measurable volumes ∼94 mm^3^ at day 10 by an external caliper.

### External Caliper

The evaluation of tumor growth using an external caliper is a widely accepted method for its ease of use. The superficial tumor volume is inferred with the assumption of an ellipsoid shape defined by the greatest longitudinal diameter (*L*, commonly termed long axis or length) and the transverse diameter defined by its width (*W*), which are determined with the caliper using the following simplified eq 3:^56,57^
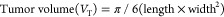
3

### Statistical Analysis

GraphPad Prism (GraphPad Software) was employed for statistical analysis using Student’s *t* test and two-way ANOVA of ellipsoid measurements of tumor volume.

## Results and Discussion

### Design for Minimized Fiber Diameter

**Figure 1:**
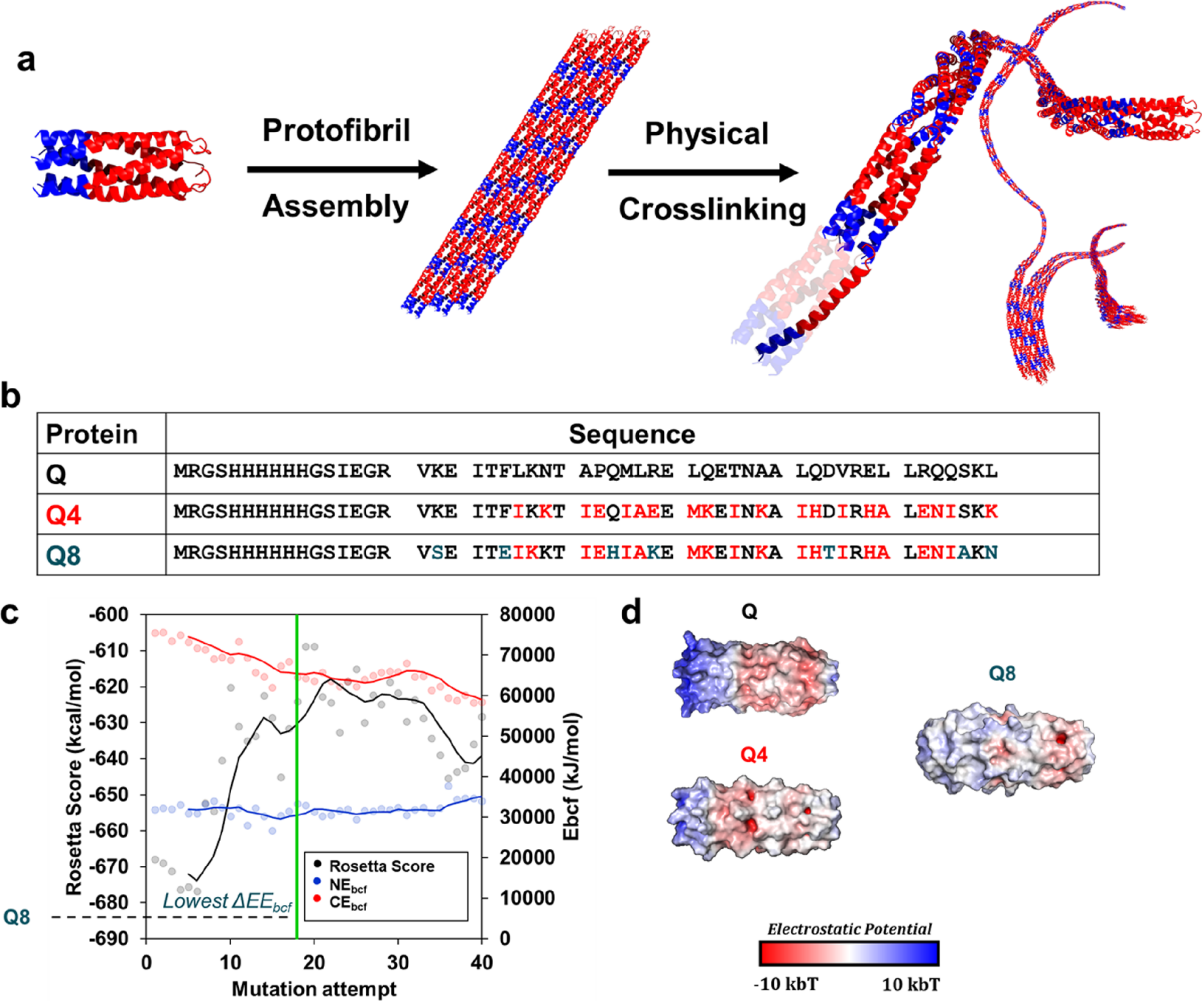
(a) Schematic of supramolecular assembly based on electrostatic potential differences of the N-terminus (blue) and C-terminus (red), which have positively and negatively charged patches, respectively. (b) Comparison of protein sequences of the original Q protein, input sequence, Q4, and the designed hydrogel sequence herein, Q8. Mutations from Q are highlighted in red. Further mutational differences from Q4 are highlighted in blue. (c) Resulting multimodal Monte Carlo search simulation used to search for Q8. (d) Comparison of electrostatic potential maps of Q, Q4, and Q8.

We set out to design a coiled-coil system with minimized fiber diameters to allow for supramolecular assembly with increased gelation kinetics (Figure 1a). In pursuit of a minimized fiber diameter while maintaining protein stability, we employed a custom trimodal Monte Carlo search, adopted from our previous characterization of coiled-coil protein nanofibers. Here, we searched for a minimum ΔEE_bcf_ based on the electrostatic potential of surface residues labeled as *b*, *c*, and *f* helical wheel positions between the C- (CE_bcf_) and N- (NE_bcf_) termini. Previously, we pursued hydrogels of varying CE_bcf_ and NE_bcf_ to better understand the sequence-function space of the Q hydrogel system. We discovered that hydrogels with lower ΔEE_bcf_ possessed smaller fiber diameters and faster gelation kinetics. We thus instead chose to modify our Monte Carlo search to minimize CE_bcf_ and NE_bcf_, where we hypothesized that their minimization would allow us to search for a coiled-coil protein sequence that possessed a low lateral supramolecular assembly behavior. We simultaneously maintained the goal of searching for a protein that possessed a minimum Rosetta score to ensure a coiled-coil super secondary structure.

Previously, our fastest gelling constructs, with the lowest electrostatic potential energy, were found to be Q4 and Q5, where Q4 was shown to have a slight improvement in UCST behavior.^29^ Thus, we used Q4 as a starting point in our Monte Carlo search, where its sequence was used as the input in five simulations (Figure 1b). Each simulation was allowed to mutate for a minimum of 150 mutations, where a minimum ΔEE_bcf_ was typically found within 30 successful mutations. The sequence for Q8 that was selected for further characterization possessed the lowest number of mutations that garnered the lowest ΔEE_bcf_ of its respective simulation (−3.1 × 10^5^ kJ/mol) and possessed a low Rosetta energy score at −624 kcal/mol (Figure 1c). Previously, the lowest ΔEE_bcf_ was found in Q5 at −4.3 × 10^5^ kJ/mol, which yielded the thinnest nanofibers with the fastest gelation rate at 22.2 ± 8.4 nm and 11.5 ± 1.5 h, respectively. The substantial decrease in surface electrostatic potential energy was also apparent in the electrostatic potential map of Q8 compared to that of Q4 and Q (Figure 1d). To generate a best pose from an equal starting point to previous Q variant models for comparison, a best scoring Q8 pose of −607 kcal/mol was generated from mutating the original Q input sequence.^29^

Yet, Q8 still possessed a high degree of sequence variance from its input with seven mutations made from the Q4 input sequence (Figure 1b). In comparison to the original Q sequence, Q8 possessed a substitution at 25 of the 38 residue sites of the coiled-coil or only a 34% homology to the Q coiled-coil sequence. The low sequence similarity between Q8 and Q highlights the ability to use the Rosetta score in conjunction with ΔEE_bcf_ to redesign protein sequences while maintaining or improving electrostatic protein–protein interaction.

### Structure and Nanoassembly

**Figure 2:**
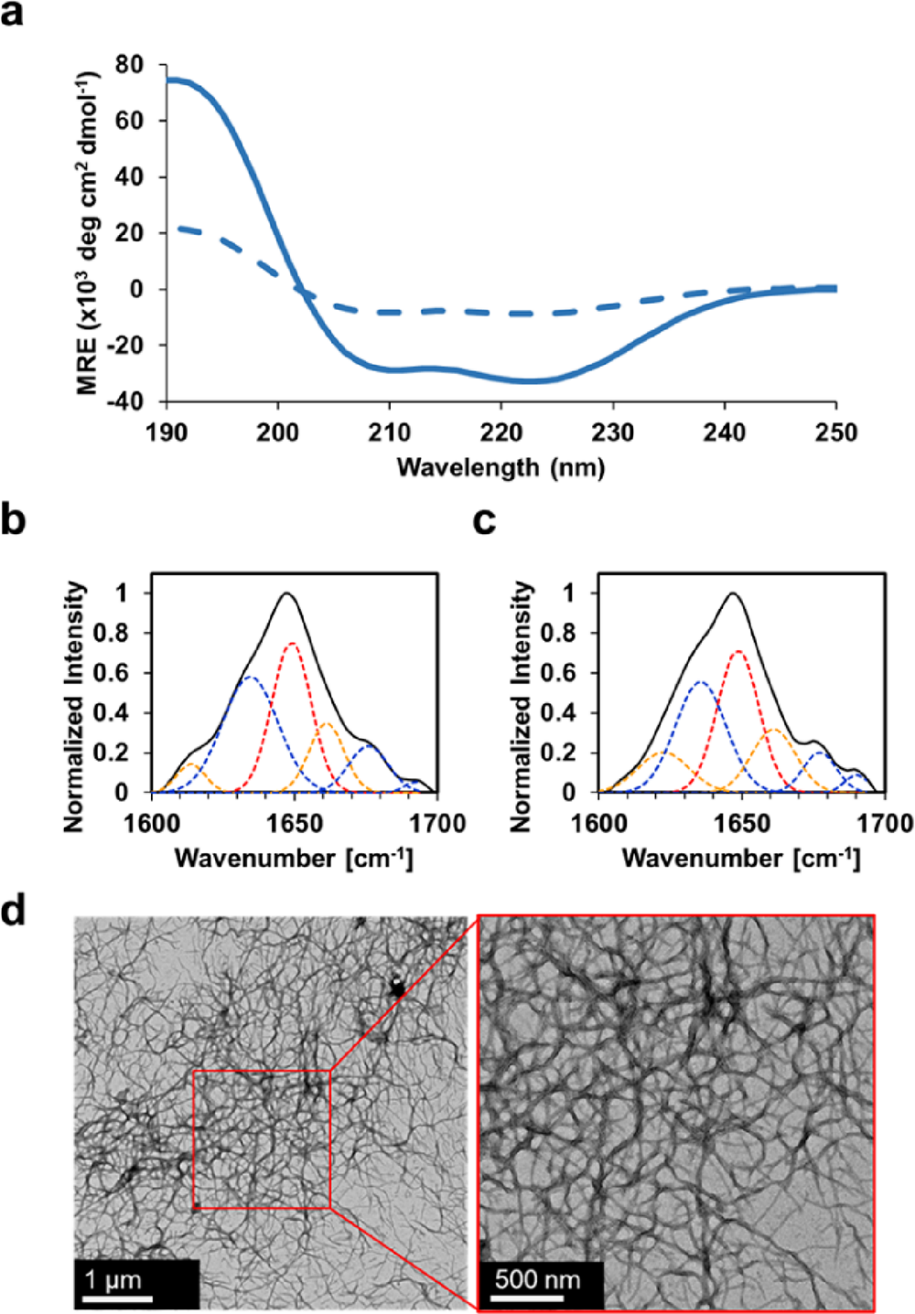
(a) Circular dichroism spectra of Q8 in the solution state (solid line) and gel state (dashed line) using a wavelength scan at 4 °C. The wavelength scan represents the average of three independent trials. ATR-FTIR spectra of the amide I bond region of Q8 in the (b) solution state and (c) gel state. (d) Representative transmission electron micrographs of the Q8 hydrogel.

The protein secondary structure was evaluated with CD and ATR-FTIR. The secondary structure characterized by CD (Figure 2a,Table S1) of Q8 in the solution phase revealed a double minimum of −27,000 ± 2000 deg·cm^2^·dmol^–1^ at 208 nm and −33,000 ± 3000 deg·cm^2^·dmol^–1^ at 222 nm, yielding a significant increase in structure compared to previously reported Q hydrogels.^29,45^ Moreover, the spectra resulted in a 222/208 ratio of 1.2 ± 0.1, where a high 222/208 ratio is indicative of a coiled-coil.^58−60^ After incubation at 4 °C, Q8 exhibited a dampening of its signal, characteristic of previous Q hydrogels, while retaining a double minima at −8000 ± 2000 deg•cm^2^•dmol^–1^ at 208 nm and −9000 ± 1000 deg•cm^2^•dmol^–1^ at 222 nm. Similarly, this resulted in a high 222/208 ratio of 1.1 ± 0.0.

The secondary structure deconvolution was assessed by attenuated total reflectance-Fourier transform infrared (ATR-FTIR) spectroscopy. A strong helical peak was confirmed by deconvolution of ATR-FTIR from the solution phase (Figure 2b,Table S1), which possessed 28.8 ± 4.2% helical content compared to the gel phase (Figure 2c,Table S1), which exhibited 34.7 ± 2.0%; this indicated a transition to a more helical structure from solution to gel. Previously, we noted a strong relationship between hydrogel helicity, structural transition, and Rosetta score. The increase in the structure of Q8 was consistent with the previous model where Q8 Rosetta score was a good predictor of structural transition from gelation, whereas using Rosetta score to predict α-helicity of Q8 was not a good predictor in this case (Table S2).

The fiber morphology and supramolecular assembly of Q8 were assessed by transmission electron microscopy (TEM). Q8 micrographs exhibited thin, physically entangled nanofibers with an average fiber diameter of 23.1 ± 4.6 nm (Figure 2d, Figure S2). Q8 exhibited one of the thinnest observed fiber diameters among our Q hydrogels. Only Q5 boasted lower fiber diameters measuring at 22.2 ± 8.4 nm, a negligible difference to Q8 (*p* value 0.35).

### Material Strength and Rheology

**Figure 3:**
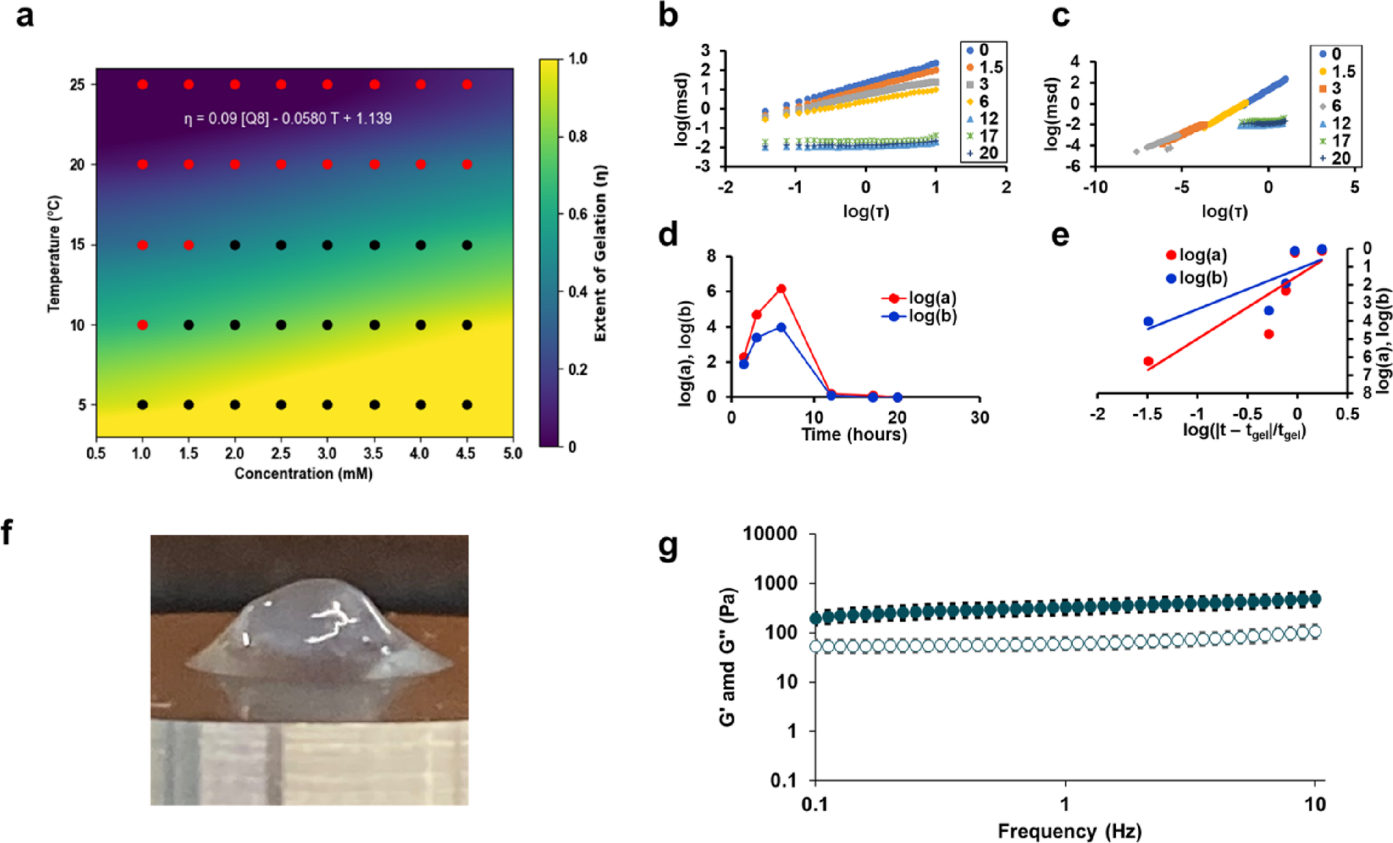
(a) Phase diagram of Q8 analyzed using bivariate regression analysis for the dependence of temperature and concentration on the solution to gel transition. Representative microrheological analysis using MPT for Q8 showing (b) log–log plot of MSD and lag time τ and (c) time-cure superposition of MSD vs τ. (d) Logarithmic shift factors for the vertical (log(a) in red) and horizontal (log(b) in blue) directions used in the time cure superposition to determine the *t*_c_. (e) Log–log plot of the shift factors and their distance from *t*_c_ determined by the ratio of the logarithmic slopes of the horizontal to vertical shift factor. (f) Representative image of Q8 in the gel state (2 mM) after loading onto a parallel plate rheometer. (g) Average storage modulus (*G*′, filled markers) and loss modulus (*G*′’, empty markers) of Q8 (2 mM) using a parallel plate rheometer at various frequencies at 4 °C. Error bars represent the standard deviation of three independent trials.

The upper critical solution temperature (UCST) behavior was assessed using tube inversion at various concentrations (1–5 mM) and temperatures (5–40 °C) (Figure 3a). Q8 exhibited a gel-like behavior at a maximum temperature of 15 °C when concentrated to 2 mM. Using a bivariate regression analysis, as done previously,^29,31^ Q8 was determined to have a UCST of 18.2 °C at 4.5 mM. In comparison, Q8 possessed a lower UCST than all previous hydrogels with the exception of Q and the lowest thermal dependence coefficient of −0.0580, indicating low hydrogel thermal stability.^29^

Q8 was assessed for gelation kinetics using passive microrheology^46^ at 2 mM and incubation at 4 °C. Using MPT, fluorescent tracer beads were tracked for their relative bead trajectories over time until negligible mean-square displacement (MSD) changes were observed using a best-fit sigmoidal analysis^46^ (Figure S3), which exhibited a critical gelation time (*t*_gel_) of 8.1 ± 0.4 h. At the beginning of the assay, Q8 exhibited an expected logarithmic slope of the particle MSD of 1.00, consistent with Brownian motion. Notably, Q8 exhibited a gelation plateau at 0.03 ± 0.02, indicative of zero movement of the tracer beads and a complete transition to viscoelastic behavior, an unprecedented extent of gelation for our Q hydrogel system. Using superposition analysis (Figure 3b–e), Q8 possessed a critical relaxation exponent (*n*_c_), which is characteristic of the degree of cross-linking of 0.62 ± 0.02, consistent with previous Q hydrogels.^29^ By superposition analysis, Q8 possessed a gelation rate faster than that of any Q hydrogel predecessor with a critical time to gelation (*t*_gel_) of 7.5 ± 1.2 h, in agreement with sigmoidal analysis. The increased gelation rate of Q8 allows for more facile treatment preparation where a sample can be expected to be ready for drug loading overnight, where previous Q hydrogels would require approximately 1 to 5 more days until the hydrogel has shown negligible changes in gelation to be ready for drug loading.^29^

Q8 confirms that small diameter fibers lend themselves to faster gelation rates (Figure S4a). However, using the ΔEE_bcf_ metric alone to predict the gelation of Q8 based on the respective model outlined previously,^29^ Q8 would possess a *t*_gel_ of −17.9 h, outlining the limits of the model at extreme ΔEE_bcf_. We have previously hypothesized that an exponential fit may be a better predictor based on the large increase in gelation time^29^ and fiber diameter^29,30^ found in Q. With the addition of Q8 at the lower limits of gelation time, an exponential model for the impact of ΔEE_bcf_ appears to be a better suited relationship (Figure S4b) and maintains a strong *R*^2^ value of 0.85. This model suggests that to design a coiled-coil capable of gelation within <1 h, ideal for *in situ* gelation, a coiled-coil design would need to possess a ΔEE_bcf_ of ∼4400 kJ/mol, indicating that this coiled-coil system is likely not capable of generating an ideal *in situ* gelator by canonical sequence modification alone.

Finally, the mechanical strength of the Q8 hydrogel was assessed using parallel plate rheometry after incubation of 2 mM concentrations at 4 °C. After removal of Q8 from its Eppendorf tube, we observed Q8 to possess a decreased extent of deformation (Figure 3f) compared to previous Q hydrogel systems such as Q (Figure S5). The increased elasticity of Q8 was confirmed in a frequency sweep where Q8 (Figure 3g) demonstrated *G*′ > *G*′′, indicative of a gel-like behavior, and a substantial increase in storage modulus and loss modulus at all frequencies. Specifically, Q8 possessed an average storage modulus (*G*′) of 490 ± 60 Pa and a loss modulus of 110 ± 12 Pa at 10 Hz, which represent a 1.7- and 5-fold increase, respectively, to the next highest modulus in Q7 previously.^29^ In particular, the large increase in loss modulus is indicative of increased resistance to deformation, suggesting the retention of entangled chains.

### Drug Loading

Because Q8 possessed an increased gelation rate and improved mechanical strength, we further characterized it for sustained small molecule delivery where we chose to investigate its ability to encapsulate and release doxorubicin (Dox) for TNBC. Drug loading in 2 and 3 mM concentrations of Q8 was tested using 1 and 3 mM Dox dosages based on the maximum tolerated dose (MTD) of Dox used in previous mouse models^61^ and clinical dose.^62^ Using a Dox standard curve (Figure S6), we determined Q8 to have a maximum loading of 1 mM inside the gel, at 2 or 3 mM Q8 protein concentrations, when applying 1 or 3 mM applications of Dox at 2× volumes indicating a saturation of 1 mM Dox regardless of protein or Dox concentrations. We attribute this Dox loading limit to the overall solubility limit of Dox within the matrix where protein content provides a negligible change in weight percent (1.3–2.0 wt/wt %) and resulting Dox–matrix interaction.

**Figure 4:**
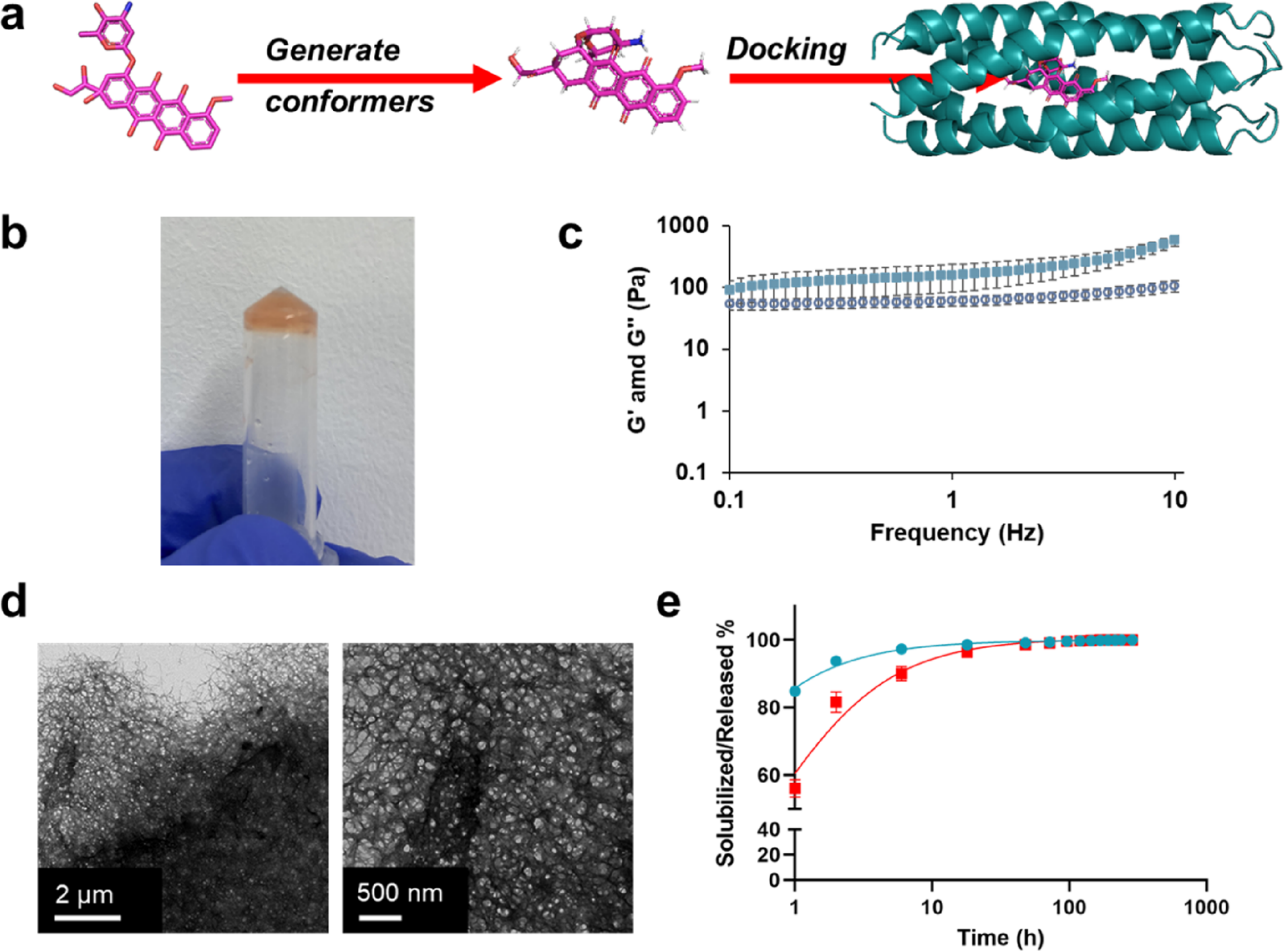
(a) Schematic of workflow for Dox docking in Q8 with the resulting best scoring pose of Q8•dox. (b) Representative photograph of Q8•Dox after tube inversion while the cells were resting at room temperature for 2 weeks. (c) Average storage modulus (*G*′, filled markers) and loss modulus (*G*′’, empty markers) of Q8•Dox after loading by syringe injection using a 26G syringe on a parallel plate rheometer at various frequencies. Error bars represent the standard deviation of three independent trials. (d) Representative transmission electron micrographs of Q8•Dox hydrogels. (e) At physiological temperature, the Q8•dox hydrogel undergoes both erosion (shown in cyan) and Dox release (shown in red). These processes are measured by tracking the cumulative release of Q8 protein solubilized from the hydrogel and the cumulative release of Dox directly from the hydrogel.

The ability for Dox to be encapsulated by Q8 was further investigated by the Rosetta macromolecular modeling suite. Dox conformers were generated by OpenEye OMEGA^44^ prior to being placed along the long hydrophobic pore of Q8 in PyMOL. Q8•Dox structures were subsequently subjected to the GALigand Docking protocol^41^ (Figure 4a). Best pose structures revealed a preference for Dox to dock in the very center of the hydrophobic pore, similar to curcumin binding in previous Q variants.^30^ Previously, Q was shown to possess an N-terminal and C-terminal binding pocket for Dox where it appeared close to the end of the protein cavity. The best docking pose of Q8•Dox revealed an interface score of −47 Rosetta energy units (REUs), indicating a strong small molecule and similar to curcumin binding in previous Q variants.^30^ Overall, the negative interface score and central position of the Dox molecule in Rosetta modeling indicate Dox to be well encapsulated in the pore of the Q8 coiled-coil.

Upon encapsulation of Dox, Q8•Dox exhibited a substantial increase in material strength, where Q8•Dox showed complete stabilization as a hydrogel and was able to pass the tube-inversion test after resting at room temperature for at least 2 weeks (Figure 4b). Because the intended application of the Q8•Dox was for subcutaneous injection, the Q8 and Q8•Dox material was loaded into a 1 mL syringe and allowed to gel prior to injection onto a parallel plate rheometer to assess the relative material strength (Figure 4c). In the case of Q8, the shearing by the syringe completely removed the viscoelastic nature of the gel, leaving it in solution. Q8•Dox, however, revealed complete stability even after shearing, exhibiting an average *G*′ of 580 ± 50 Pa and *G*′′ of 60 ± 20 Pa. Despite the addition of shearing, we note that Q8•Dox indeed possessed a slightly greater *G*′ than Q8 alone without shearing, indicating that the protein–small molecule combination results in a more densely cross-linked material.

Improved physical cross-linking was visually assessed by TEM (Figure 4d, Figure S7). Q8•Dox resulted in a dense bed of cross-linked nanofibers. Additionally, fibers appeared to bundle together to a greater extent, increasing the average fiber diameter to 30.1 ± 14.0 nm. The substantial difference in dispersity of the fibers in the micrographs suggested that Dox acts as an additional cross-linking point in addition to its encapsulation in the coiled-coil pore, similar to other small hydrophobic molecules before it in our coiled-coils such as curcumin (CCM).^31,45^ Thus, the greater relative increase in storage modulus is explained by increased cross-linking of the fibers induced by Dox.

To quantify the relative stability of Q8•Dox for physiological drug delivery, we employed an erosion and solubilization experiment (Figure 4e) as done previously for our CCM-bound coiled-coils.^30^ Here, the erosion of protein and release of Dox were measured at interval times until negligible increases were observed. Consistent with CCM-bound Q release,^45^ Q8•Dox demonstrated Q8 to solubilize faster than Dox. The outpaced release of the protein over its encapsulated small molecule can be attributed to Dox encapsulation within the pore of the coiled-coil, confirmed by Rosetta docking simulations (Figure 4a), which is not dependent on the hydrogel matrix degradation. In comparison, Q8 alone exhibited immediate solubilization, similar to Q previously.^45^ Q8 and Dox exhibited complete solubilization from Q8•Dox within approximately 18 h and exhibited a burst-like release (Figure 4e) dominated by diffusion.^63^ Overall, Q8•Dox possessed an increased rate of release compared to Q•CCM,^45^ which required approximately 2 weeks to fully release. To note, our release study was performed using our standard buffer for Q variants, i.e., 50 mM TrisHCl and 500 mM NaCl (pH 8.0), which may not reproduce similar release kinetics in physiological conditions, and we defer to our *in vivo* release study *vida infra*. However, Tris buffers have been used in several FDA-approved formulations and are considered effective to stabilize pH in the physiological range,^64^ an important consideration for our pH-sensitive Q hydrogel system.^47^

### *In Vivo* Mouse Study for Tumor Response to Treatment

To assess the impact of Q8•Dox on the tumor response, tumors derived from the 4T1 cell line were used. Briefly, the 4T1 cell line, which is derived from a spontaneous tumor in a BALB/c mouse, is a widely used and invaluable model for studying breast cancer metastasis, particularly TNBC.^65^ These cells closely mimic the aggressive behavior of human TNBC, making them a crucial tool for developing and testing new therapeutic strategies.

**Figure 5:**
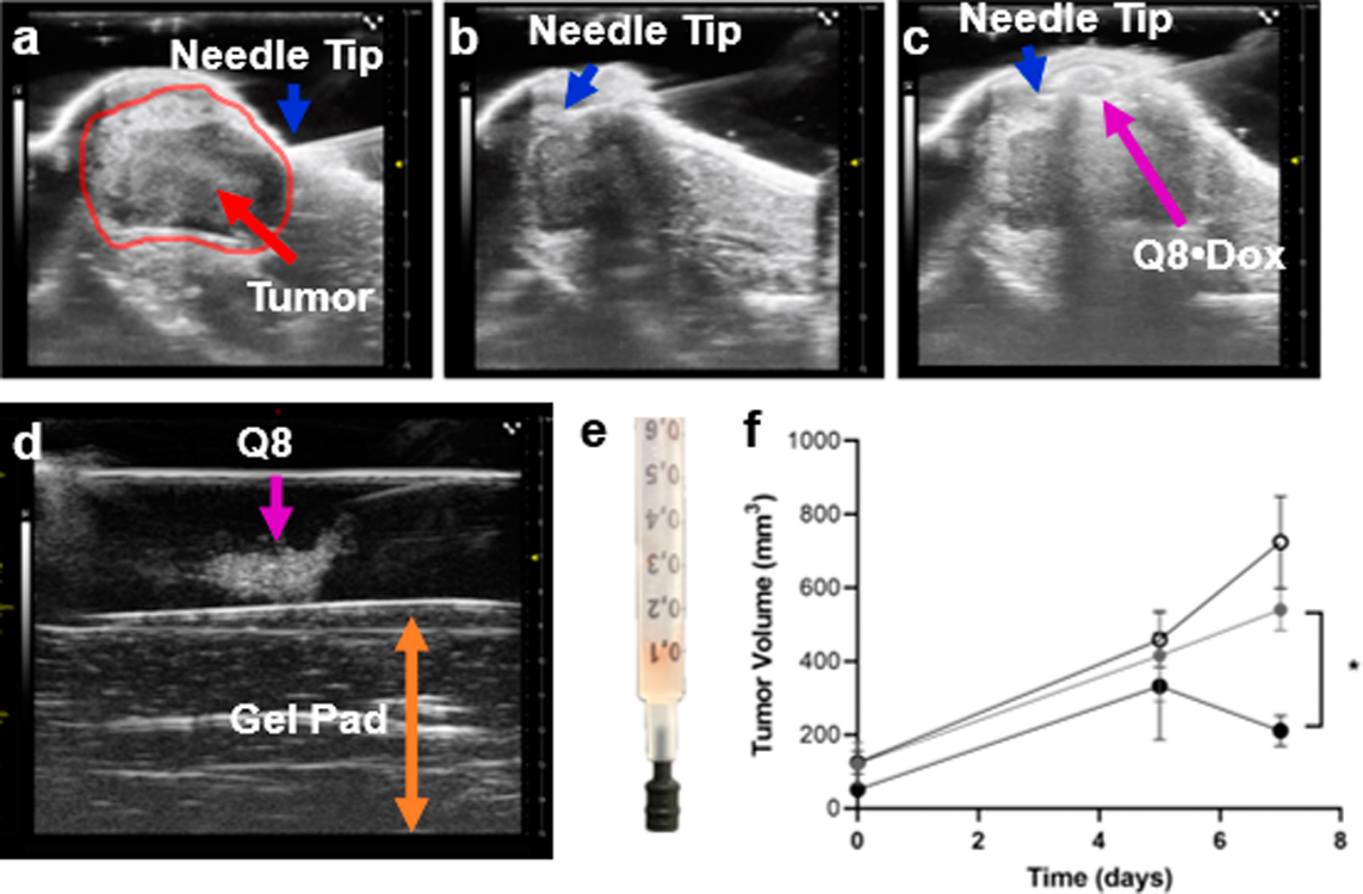
Ultrasound-guided injection. (a) Axial ultrasound view of the lower abdomen near the fat pad before needle insertion. The needle is tilted at 45° for easy intracutaneous insertion and delivery over the tumor. (b) Successful intracutaneous needle placement prior to injection. (c) Successful injection of Q8•Dox onto the tumor appeared as an echogenic high-frequency ultrasound signal surrounded by a hyperechoic rim. The blue arrow indicates the syringe tip, and the magenta arrow indicates the presence of echogenic Q8 from Q8•Dox. (d) Phantom ultrasound image of the Q8 hydrogel injected into ultrasound gel. The B-mode image clearly shows the hyperechoic signal from Q8 (magenta arrow) enhancing image contrast. (e) Representative photograph of Q8•Dox treatment loaded into a 1 mL syringe, gelled after 48 h at 4 °C, and followed by Dox loading and washing. (f) Average tumor volume following treatment of Q8 hydrogel (black open circles), Dox (gray filled circles), and Q8•Dox (black filled circles), measured at baseline (0 days) and at 5 and 7 days post-treatment. Error bars represent the standard error of five independently treated mice in each group. A figure with standard error for the Q8•Dox and Dox group and four mice for the Q8 group is shown in Figure S7. *p value < 0.05 calculated by two-way ANOVA.

Image-guided intracutaneous injection was used for aseptic and reproducible positioning and delivery of the Q8 compound onto the tumor area, which also highlighted the noninvasive administration of Q8•Dox. This approach facilitated real-time monitoring of the injection process and ensured optimal subsequent localized release of doxorubicin. Consistent with our recent work using a coiled-coil fiber to target disease prevention in osteoarthritis,^66^ we used the same approach for localized injection and release followed more recently with delivery via high-frequency ultrasound image guidance for noninvasive monitoring of the fate and degradation of protein structure using multimodal imaging.^38^ In this study, we focused on the intracutaneous delivery of the Q8 hydrogel using high-frequency ultrasound over the tumor. Afterward, volumes of the tumors were monitored by caliper measurements over the course of the treatment. Importantly, the Q8 hydrogel appeared immobilized because of the echogenic properties monitored noninvasively *in vivo* with high-frequency ultrasound (Figure 5a–c).

An axial ultrasound image view of the mouse abdomen clearly reveals distinct echogenic multilayers (Figure 5a). The outermost thin, bright line is the skin, highly reflective (hyperechoic) of ultrasound waves. Below lies the thicker, darker fat pad, consisting of adipocytes whose lipid droplets scatter ultrasound energy, forming a less reflective (hypoechoic) layer and casting a shadow. Within this fatty region, the implanted breast cancer tumor, delineated by the red line, appears as an irregular, hypoechoic area, disrupting the fat pad’s uniform darkness. Its margins might be indistinct, blending with the surrounding lipid, or sharply defined, indicating its infiltrative nature. Internal heterogeneity hints at its character, with anechoic pockets suggesting necrosis and brighter areas possibly indicating denser regions within the tumor.

The precise needle placement for the injection is visualized (Figure 5b). A 30G needle is carefully inserted laterally within the intracutaneous space above the tumor mass region, with its tip depicted by the blue arrow. The presence of the Q8 hydrogel (3 mM, 100 μL, depicted by the magenta arrow) is observed following slow infusion (Figure 5c), appearing as a hypoechoic signal surrounded by a bright rim. Echogenicity of the Q8 hydrogel is initially characterized with a phantom setup (Figure 5d), which is reproduced during *in vivo* experiments (Figure 5c).

To assess the impact relative to Dox injections alone, Dox was prepared as 1 mM in 100 μL of 50 mM Tris and 500 mM NaCl (pH 8.0) and 5% v/v DMSO. To achieve uniform biodistribution of Dox throughout the mouse body with effective first-pass arterial circulation, we opted for intracardiac injection into the left ventricle. Although seemingly invasive, this intracardiac approach demonstrates high reproducibility across animals, robust safety, and reduced animal discomfort compared to that of open surgery, which otherwise would have been necessary to achieve similar consistency. Extensive experience with intracardiac injections confirms rapid mouse recovery without observable morbidity.^55^ In contrast, tail injections, although commonly used, pose challenges in achieving reproducible drug administration due to their dependence on several factors, including the mouse strain, age, health status, and researcher skill. This dependence inherently leads to an increased variability. Tumor volumes were assessed prior to treatment (day 0) and after treatment (days 5 and 7) within a week of subcutaneous injection of Q8•Dox, Q8, or intracardial injection of Dox. Intravenous or intracardial injection of Dox was well-established as the standard administration method due to its superior performance, whereas local administration of Dox is not effective for tumor regression due to high diffusion.^67^ Thus, a single intracardial injection of Dox was used at an approximate dosage of 3 mg/kg using 1 mM per 100 μL of intracardial injection. For reference, the maximum tolerated dose (MTD) in mice is 10 mg/kg,^61^ and 1.9 mg/kg is used clinically.^62^ Q8•Dox was applied with an equivalent dosage of 3 mg/kg at an equivalent 1 mM Dox per 100 μL of subcutaneous injection. As a control, 100 μL of Q8 was employed. To prepare Q8 and Q8•Dox treatments, 1 mL disposable syringes (American Health Service) were loaded with Q8 hydrogel and incubated at 4 °C for 2 days to allow for gel formation while using the rubber stopper of another disposable syringe as a temporary cap (Figure 5e). Dox was allowed to diffuse into the hydrogel matrix of Q8•Dox samples for 24 h before removal and washing with two rounds of buffer, consistent with previous Q8•Dox preparations.

Following 7 days of treatment, Q8•Dox exhibited superior tumor suppression compared to both treatments of Q8 and Dox alone (*p* values of 0.02 and 0.07, respectively; Figure 5f). Notably, the effect on tumor volume only became apparent at day 7. This delayed response is interesting given that *in vitro* release studies showed near complete solubilization of Q8•Dox within the first 18 h. This suggests that improved solubility and sustained release within this initial window are sufficient to provide a lasting impact on tumor suppression at later stages of growth. A similar delayed response was also noted in LCST Dox-bound PLGA-*b*-PEG-*b*-PLGA hydrogels combined with clay nanodisks that provided similar burst release kinetics yet only demonstrated tumor suppression by day 7 of treatment.^68^ Subcutaneous injection of Q8•Dox resulted in a relative tumor shrinkage of −36%, whereas Q8 and Dox alone led to relative growth of 58 and 30%, respectively, between days 5 and 7.

Importantly, tumor growth at later stages, particularly in the Q8-treated group, exhibited high variability. One Q8-treated tumor deviated considerably from the group pattern, measuring 1200 mm^3^, significantly beyond the expected range (600 ± 140 mm^3^). Given this exceptional case and the known heterogeneity of tumor growth,^69^ we have provided analysis with the outlying tumor excluded to ensure a more robust and consistent representation of the group’s response to treatment. We have provided a visualization of the data excluding this outlier in Figure S8. The Q8•Dox-treated group showed a significant decrease in tumor volume at day 7 compared to both Dox- and Q8-treated groups (*p* value < 0.05, Figure S8**).**

## Conclusions

We present a novel coiled-coil protein, Q8, engineered through a multistate Monte Carlo search algorithm. Q8 features minimized fiber diameters, leading to an enhanced gelation rate and mechanical strength. Its design embodies recent discoveries in the functional design of supramolecular protein assemblies and electrostatic protein–protein interactions, showcasing its potential for biomedical applications. Notably, Q8 exhibits efficient encapsulation and release of chemotherapeutic Dox, making it a promising drug delivery vehicle for TBNC treatment. Moreover, Dox-bound Q8 (Q8•Dox) enables echogenic monitoring for precise subcutaneous injection, facilitating localized tumor therapy. Remarkably, a single subcutaneous injection of Q8•dox effectively shrinks tumors compared to intracardial Dox after just 1 week.

A previous work has also highlighted the use of thermosenstive hydrogels for subcutaneous delivery of doxorubicin for tumor treatment.^70^ Specifically, hydrogels composed of PLGA-*b*-PEG-*b*-PLGA hydrogels combined with clay nanodisks and Dox have shown to be effective after a single injection as well.^68^ These hydrogels, however, do not offer sequence-based modularity as a protein-based hydrogel, and instead, its lower critical solution temperature behavior is modulated by introduction of copolymers and intercalated molecules.^68,71^ Interestingly, the Dox-bound PLGA-*b*-PEG-*b*-PLGA hydrogels exhibit many similar behaviors to Q8•Dox including decreased pore sizes upon Dox binding, similar burst release times, and similar tumor suppression using single ∼100 nmol dosages.^68^ Dox-bound PCL-PEG-PCL thermosensitive hydrogels are similarly designed for sustained drug release; however, effectiveness *in vivo* has not been confirmed.^72^ Notably, other hydrogel or nanogel systems have also been generated for the potential delivery of other anticancer agents.^70,73^ In contrast to synthetic polymer systems, the design of a protein-based hydrogel demonstrates the remarkable potential of computational design to engineer sequence-modular biomaterials for therapeutic applications.

## Supporting Information Available

Figures of SDS-PAGE for purification of Q8, TEM of Q8 and Q8•Dox at various resolutions, sigmoidal fit of Q8 microrheological exponents, updated linear fits based on correlation for average fiber diameter and calculated electrostatics with time to gelation, standard curve of absorbance at 490 nm used to measure doxorubicin concentration, representative macroscopic image of Q hydrogel, and average tumor volume including outlier in Q8-treated group; tables for secondary structure content measured by CD and ATR-FTIR of Q8 and comparison of predicted vs actual structural metrics based on previous computational models for coiled-coil secondary structure (PDF)
