# Supplementary material for: Coiled-Coil Protein Hydrogels Engineered with Minimized Fiber Diameters for Sustained Release of Doxorubicin in Triple-Negative Breast Cancer
Source: ACS Biomater Sci Eng. 2024 Apr 15;10(5):3425–37. doi: 10.1021/acsbiomaterials.4c00349 (PMC11094684; doi:10.1021/acsbiomaterials.4c00349)
Supplement: Supplementary file 1 — ab4c00349_si_001.pdf [file ab4c00349_si_001.pdf]

# Supporting Information

# Coiled-coil protein hydrogels engineered with minimized fiber diameters for sustained release of doxorubicin in triple negative breast cancer

*Dustin Britton*[†], *Jakub Legocki*[†], *Deven Paul*[†], *Olga Katsara*[‖], *Orlando Aristizabal*[§,¶], *Neelam Pandya*[§,¶], *Orin Mishkit*[§,¶], *Yingxin Xiao*[†], *Matias Aristizabal*[§,¶], *Neha Rahman*[§,¶], *Robert Schneider*[‖,#], *Youssef Z. Wadghiri*[§,¶,*], *and Jin Kim Montclare*[†,¶,∇,°,*]

† Department of Chemical and Biomolecular Engineering, New York University Tandon School of Engineering, Brooklyn, New York, 11201, USA

‖. Department of Microbiology, New York University Grossman School of Medicine, New York, New York, 10016, USA

§. Center for Advanced Imaging Innovation and Research (CAI$^2$R), New York University Grossman School of Medicine, New York, New York, 10016, USA

¶. Bernard and Irene Schwartz Center for Biomedical Imaging, Department of Radiology, New York University Grossman School of Medicine, New York, New York, 10016, USA

£ Department of Biomedical Engineering, State University of New York Downstate Medical Center, Brooklyn, New York, 11203, USA

¥ College of Medicine, State University of New York Downstate Medical Center, Brooklyn, New York, 11203, USA

# Department of Radiation Oncology, New York University Grossman School of Medicine, New York, New York, 10016, USA

∇ Department of Chemistry, New York University, New York, New York, 10012, USA

° Department of Biomaterials, New York University College of Dentistry, New York, New York, 10010, USA

* Corresponding authors

Email: montclare@nyu.edu and Youssef.zaimwadghiri@nyulangone.org

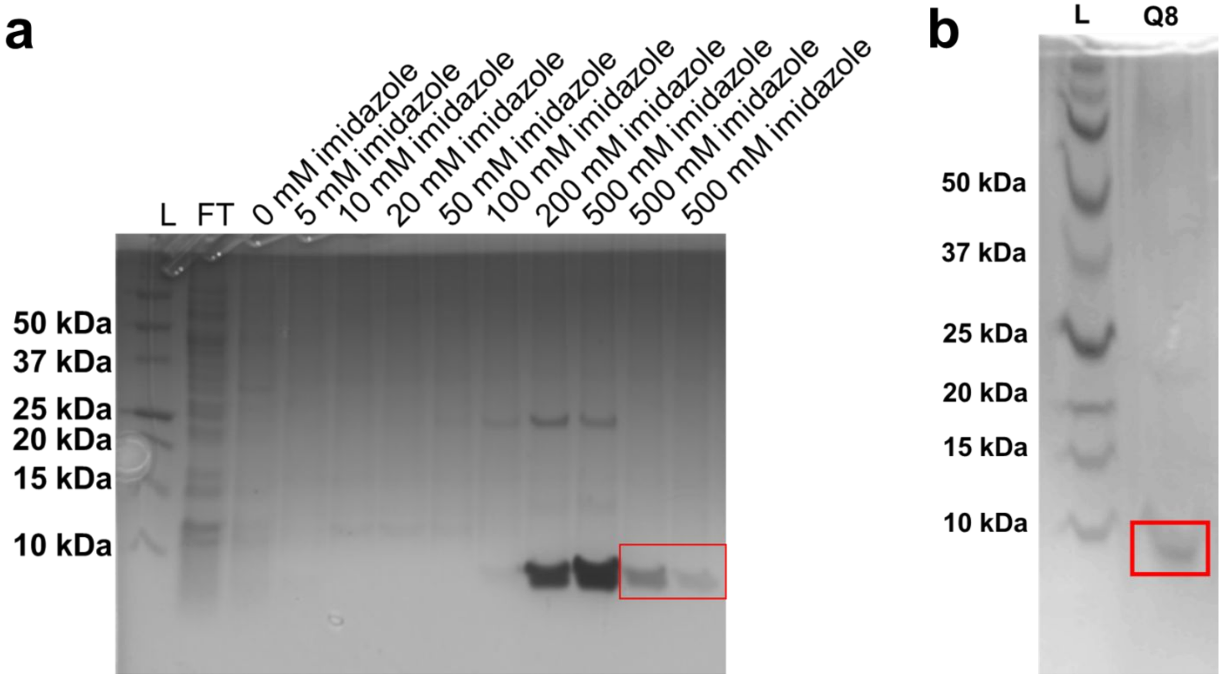


**Figure S1 a.** 12% SDS-PAGE of Q8 purification using cobalt-charged NiNTA affinity chromatography. L: Ladder, FT: flow-through. **b.** 12% SDS-PAGE of Q8 at 1 μM following dialysis and concentration.

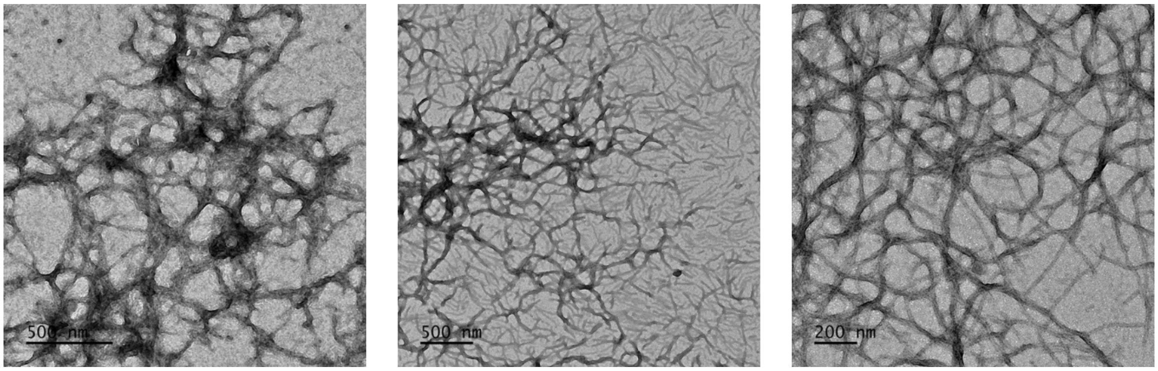


**Figure S2** Various transmission electron micrographs of Q8.

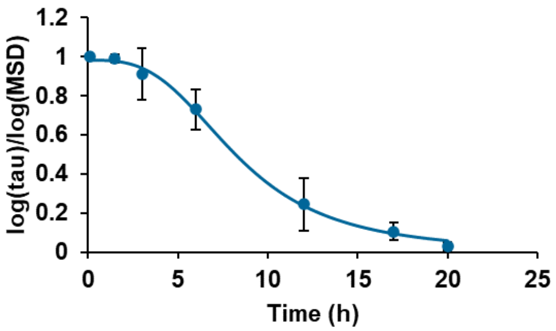


**Figure S3** Sigmoidal fit of exponents from MPT analysis of Q8 microrheological assay. Error bars represent the standard deviation of three independent trials.

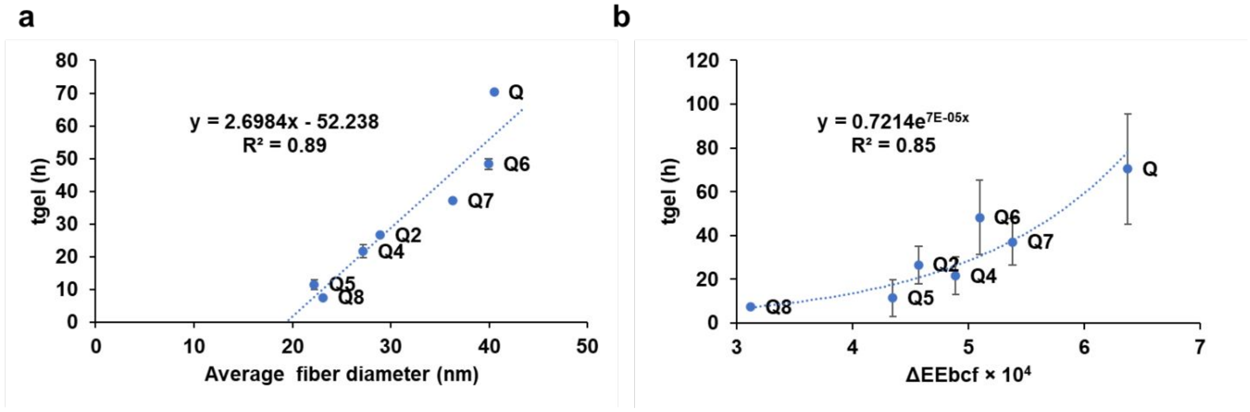


**Figure S4 a.** Updated linear regression model for relationship between average fiber diameter and critical gelation time ($t_{gel}$) for all gelling Q variants. Error represents the average of three independent trials. **b.** Exponential relationship between $\Delta EE_{bcf}$ and critical gelation time ($t_{gel}$) for *in silico* prediction. Error bars represent the standard deviation of three independent trials.

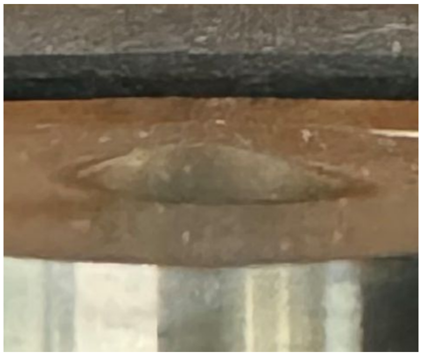

**Figure S5** Representative image of Q in the gel state (2 mM) after loading onto parallel plate rheometer.

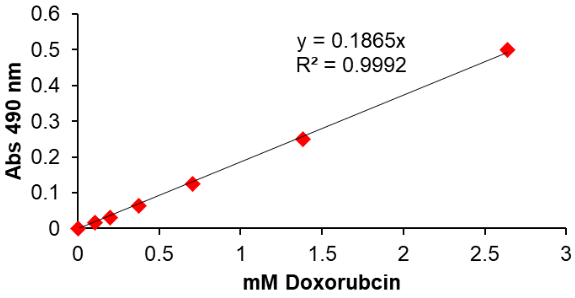


**Figure S6** Standard curve of absorbance at 490 nm by a microplate reader (BioTek) for doxorubicin concentration.

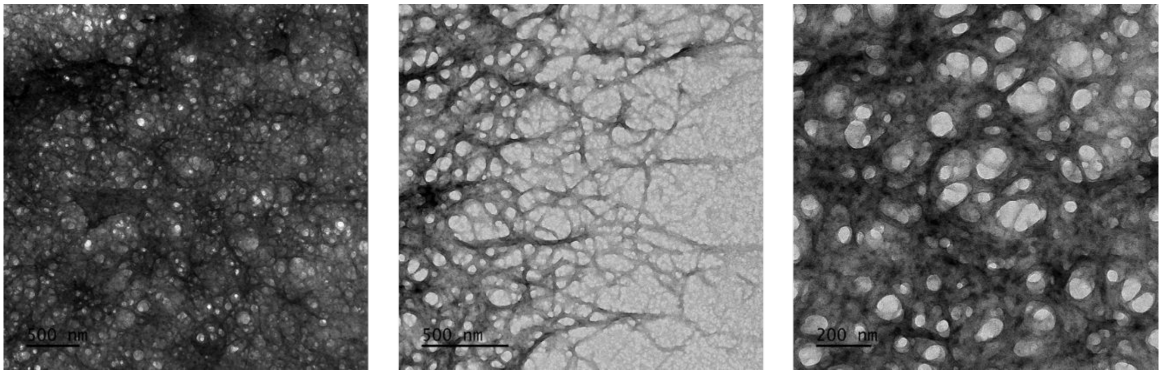


**Figure S7** Various transmission electron micrographs of Q8•Dox.

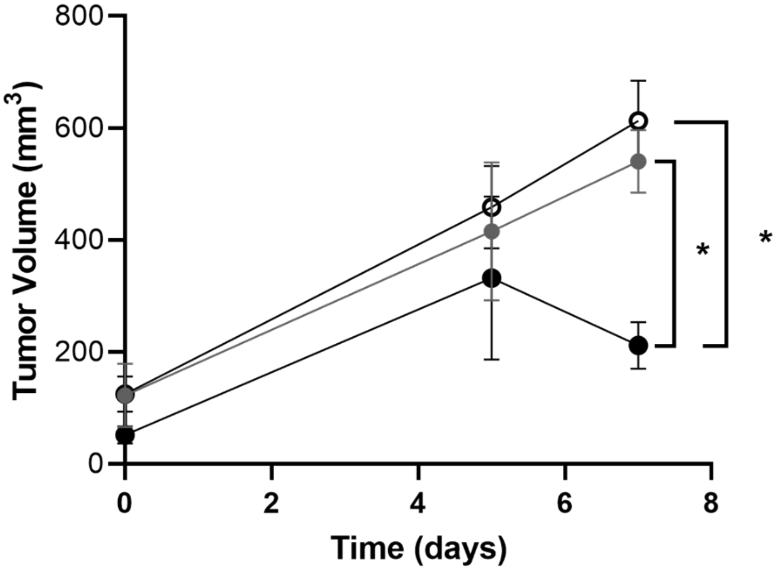


**Figure S8** Average tumor volume following treatment of Q8 (open circles), Dox (grey filled circles), and Q8•Dox (black filled circles) measured at baseline (0 days), 5 days, and 7 days post-treatment. Error bars represent the standard error of the mean: 5 mice for Q8•Dox and Dox, 4 mice for Q8. * represents p-value < 0.05 calculated by 2-way ANOVA.

**Table S1** Average and standard deviation of MRE values of CD spectra α-helical minima and % secondary structure content assessed by peak deconvolution of ATR-FTIR spectra

| Phase | Circular Dichroism Minima | | | FTIR Deconvolution (%) | | |
|---|---|---|---|---|---|---|
| | $-\Theta_{208}$ (deg•cm$^2$•dmol$^{-1}$) | $-\Theta_{222}$ (deg•cm$^2$•dmol$^{-1}$) | $\Theta_{222}$ / $\Theta_{208}$ | α-helix | β-sheet | Random Coil |
| **Solution** | 27,000 ± 2,000 | 33,000 ± 3,000 | 1.2 ± 0.1 | 28.8 ± 4.2 | 41.9 ± 5.8 | 25.9 ± 6.8 |
| **Gel** | 8,000 ± 2,000 | 9,000 ± 1,000 | 1.1 ± 0.0 | 34.7 ± 2.0 | 39.9 ± 6.7 | 26.5 ± 9.0 |

**Table S2** Comparison of experimental and predicted values of α-helicity (gel state) and increase in structured content measured by FTIR using univariate linear regression models from Britton *et al.* [1]

| Structure metric | Actual | Predicted from Model | Residual | RMSE from model |
|---|---|---|---|---|
| **α-helicity (gel) – univariate with Rosetta Score** | 34.7 | 41.6 | 6.9 | 1.0 |
| **Increase in Structure (%) – univariate with Rosetta Score** | 5.9 | 5.6 | 0.3 | 1.0 |

## Supporting Information References

(1) Britton, D.; Christians, L. F.; Liu, C.; Legocki, J.; Xiao, Y.; Meleties, M.; Yang, L.; Cammer, M.; Jia, S.; Zhang, Z.; et al. Computational Prediction of Coiled–Coil Protein Gelation Dynamics and Structure. *Biomacromolecules* **2024**, *25* (1), 258-271. DOI: 10.1021/acs.biomac.3c00968
